# Research trends in lipid-lowering therapies for coronary heart disease combined with hyperlipidemia: a bibliometric study and visual analysis

**DOI:** 10.3389/fphar.2024.1393333

**Published:** 2024-05-17

**Authors:** Quankai Cheng, Jingjing Sun, Haicheng Zhong, Ziming Wang, Chang Liu, Sheng Zhou, Jie Deng

**Affiliations:** ^1^ Department of Cardiology, The Second Affiliated Hospital of Xi’an Jiaotong University, Xi’an, China; ^2^ Department of Respiratory and Critical Care Medicine, The Second Affiliated Hospital of Xi’an Jiaotong University, Xi’an, China

**Keywords:** lipid-lowering therapy, coronary heart disease, hyperlipidemia, VOSviewer, CiteSpace, visualization analysis

## Abstract

**Background:**

Cardiovascular disease (CVD) poses a significant global health and economic challenge, with atherosclerosis being a primary cause. Over the past 40 years, substantial research has been conducted into the prevention and reversal of atherosclerosis, resulting in the development of lipid-lowering agents such as statins and fibrates. Despite the extensive literature and formulation of numerous therapeutic guidelines in this domain, a comprehensive bibliometric analysis of the current research landscape and trends has not been performed. This study aimed to elucidate the evolution and milestones of research into lipid-lowering treatments for coronary heart disease (CHD) in conjunction with hyperlipidemia through bibliometric analysis, offering insights into future directions for treatment strategies.

**Methods:**

This study examined publications from 1986 to 2023 retrieved from the Web of Science database (Core Collection). Utilizing tools such as VOSviewer, Pajek, and CiteSpace, we analyzed publication and citation numbers, H-indexes, contributions by countries and institutions, authorship, journal sources, and keyword usage to uncover research trajectories and areas of focus.

**Results:**

Our analysis of 587 publications revealed a recent surge in research output, particularly post-2003. The American Journal of Cardiology published the highest number of studies, with 40 articles, whereas Circulation received the highest number of citations (6,266). Key contributors included the United States, Japan, and China, with the United States leading in citation numbers and the H-index. Harvard University and Leiden University emerged as pivotal institutions, and Professors J. Wouter Jukema and Robert P. Giugliano were identified as leading experts. Keyword analysis disclosed five thematic clusters, indicating a shift in research towards new drug combinations and strategies, signaling future research directions.

**Conclusion:**

The last 4 decades have seen a notable rise in publications on lipid-lowering therapies for CHD and hyperlipidemia, with the United States retaining world-leading status. The increase in international collaboration aids the shift towards research into innovative lipid-lowering agents and therapeutic approaches. PCSK9 inhibitors and innovative combination therapies, including antisense oligonucleotides and angiopoietin-like protein 3 inhibitors, provide avenues for future research, intending to maximize the safety and efficacy of treatment approaches.

## 1 Introduction

**FIGURE 1:**
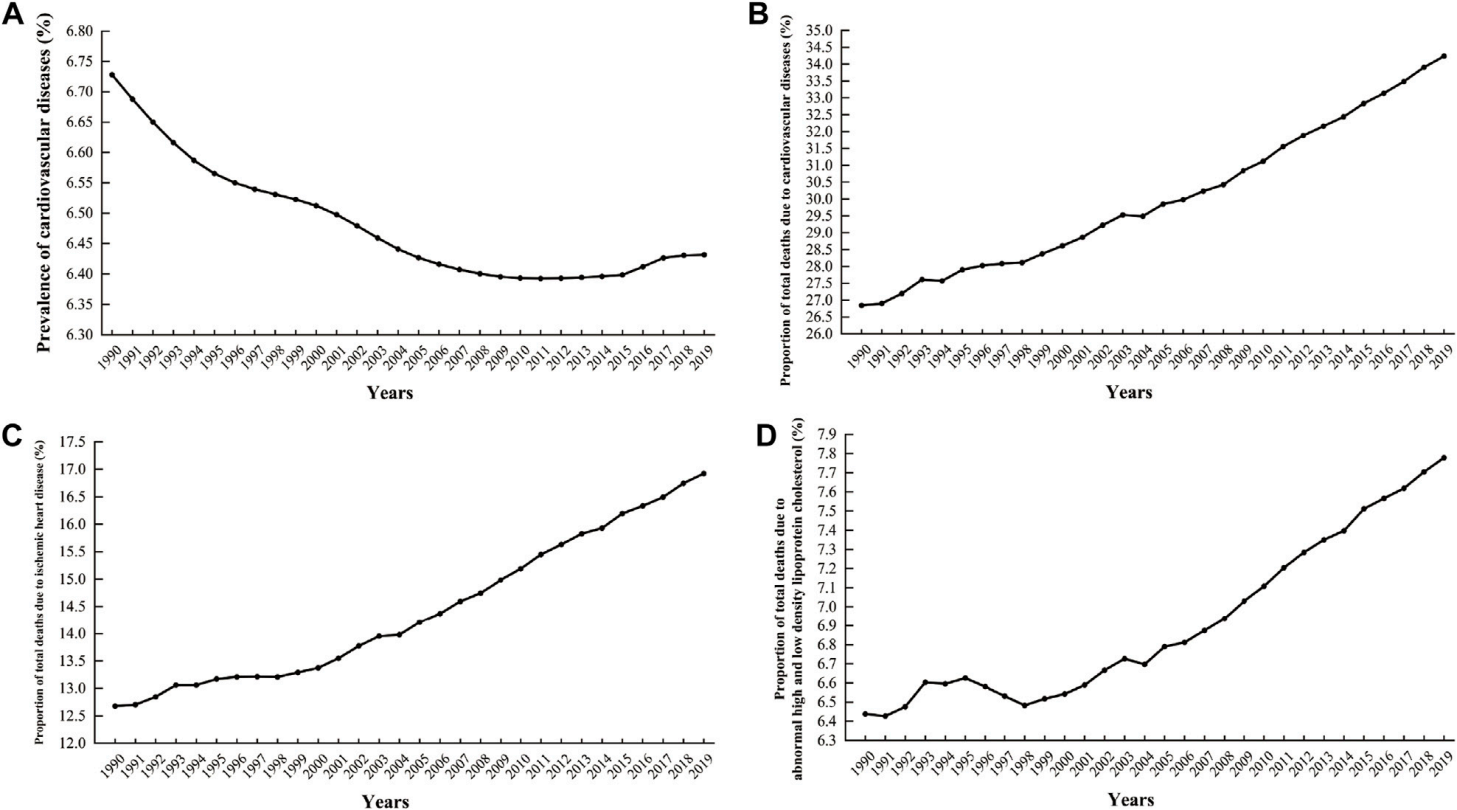
Global cardiovascular disease prevalence and associated deaths as a percentage from 1990 to 2019. **(A)** Prevalence rate of cardiovascular diseases; **(B)** The proportion of deaths attributable to cardiovascular diseases out of the total number of deaths; **(C)** The proportion of deaths attributable to ischemic heart disease out of the total number of deaths; **(D)** The proportion of deaths affected by abnormalities in high-density lipoprotein cholesterol and low-density lipoprotein cholesterol relative to the total number of deaths (Data sources: IHME, Global Burden of Disease Study (2019)—processed by Our World in Data).

Cardiovascular disease (CVD) imposes a significant global health and economic burden, contributing to approximately one-third of all deaths; coronary artery disease (CAD) is the most common form of CVD (Nowbar et al., 2019; Stone et al., 2023; Tsao et al., 2023). As shown in Figure 1A, although the overall prevalence of CVD decreased from 1990 to 2019, there has been an upward trend since 2011. Over the same period, the proportion of deaths attributed to CVD, CAD, and abnormalities in high-density lipoprotein cholesterol (HDL-C) and low-density lipoprotein cholesterol (LDL-C) have increased consistently year over year, as depicted in Figures 1B–D (Cesare et al., 2022; Mensah et al., 2023; Martin et al., 2024; Naghavi et al., 2024). CAD is caused by atherosclerosis, with risk factors including hypertension, diabetes mellitus, and hyperlipidemia (Ference et al., 2017). Among them, a direct and positive correlation exists between blood lipid levels and cardiovascular risk (Joseph et al., 2017), which may be influenced by circadian disruption (Het Panhuis et al., 2023).

Hyperlipidemia is widely recognized as a key contributor to coronary atherosclerosis, with cholesterol-rich lipoproteins accumulating in areas of the arterial intima prone to plaque formation (Skålén et al., 2002; Grundy et al., 2019). Lipids, including cholesterol and triglycerides (TG), form lipoproteins in the bloodstream because of their insolubility in aqueous environments. These lipoproteins, including very low-density lipoproteins, low-density lipoproteins (LDL), and high-density lipoproteins (HDL), constitute a crucial group of proteins (Soppert et al., 2020). Numerous studies have identified LDL-C as the most significant risk factor for the progression of atherosclerotic CVD (ASCVD), with other lipids, such as TG and triglyceride-rich lipoproteins (TRLs), also identified as risk factors for ASCVD. These findings are supported by observations made in patients with hyperlipidemia and ASCVD who maintain well-controlled levels of LDL-C (Kannel et al., 1961; Lewington et al., 2007; Miller et al., 2011; Nordestgaard, 2016a; Ganda et al., 2018; Toth et al., 2020). In patients with chronic kidney disease, the levels of plasma sphingolipids have been shown to impact the development and progression of CVD (Lidgard et al., 2023), possibly due to their role in promoting inflammatory responses in the vascular endothelium, accelerating the formation of atherosclerotic plaques, and diminishing their stability (Kugiyama et al., 1999; Karpe et al., 2001; Imke et al., 2005; Quispe et al., 2015). These effects are more pronounced in diabetic and insulin-resistant patients and might be responsible for the rapid progression of atherosclerosis in these individuals, making plasma sphingolipids potential prognostic indicators and therapeutic targets (Ginsberg et al., 2010; Toth et al., 2020). Kugiyama et al. suggested that fasting serum levels of residual lipoproteins could be used to predict coronary events in patients with CAD (Kugiyama et al., 1999). Di Angelantonio et al. concluded that risk assessments can be performed using factors such as total cholesterol, TG, and LDL-C (Di Angelantonio et al., 2012). Parish et al., 2012 demonstrated that the LDL-C particle size and content were strongly associated with major occlusive vascular events. Similarly, Mortensen et al. established a link between LDL-C levels and ASCVD events (Mortensen et al., 2023). Consequently, international guidelines universally recommend measuring LDL-C and TG levels to diagnose hyperlipidemia and predict CVD risk, indicating the preventive and therapeutic potential of reducing LDL-C and TG levels in the development and progression of coronary heart disease (CHD) and hyperlipidemia (Grundy et al., 2019; Visseren et al., 2021; Hong et al., 2023).

As research in this field intensified, the first statin lipid-lowering drug, lovastatin, became available in the United States in 1987. Subsequently, other drugs, such as fibrates and omega-3 fatty acids, emerged alongside a growing body of evidence of their critical role in lipid management for patients with CAD (Abifadel et al., 2009). However, only 23.9% of individuals at a very high risk of dyslipidemia achieved a reduction in LDL-C levels with statins alone (Yan et al., 2018). In addition, prolonged treatment with high doses of statins can cause tolerance issues and the development of secondary conditions (Al-Lamee et al., 2019; Almeida and Budoff, 2019; Dawson et al., 2022; Doenst et al., 2022). Statins increase the risk of new-onset diabetes, potentially due to a reduction in insulin sensitivity. The management of patients with diabetic dyslipidemia, therefore, requires careful consideration (Khankari et al., 2022). If pharmacotherapy is unsuccessful, surgical interventions are required, underscoring the urgent need for innovative drug discovery (Epstein et al., 2013; Al-Lamee et al., 2019). Consequently, researchers have investigated novel treatment strategies. In 2003, a collaborative team of researchers from Canada and France discovered proprotein convertase subtilisin/kexin 9 (PCSK9), leading to the development and approval of four drugs targeting this enzyme (Bao et al., 2024) and providing crucial insights into novel potential avenues for the treatment and prevention of CHD and hyperlipidemia.

Bibliometric analysis, a sophisticated statistical approach utilizing public literature databases, enables scrutinization and elucidation of evolving trends and research dynamics within specific fields (Tran et al., 2018). This method provides researchers with information on the volume and caliber of the existing literature at institutional and regional levels and forecasts future directions for scientific exploration. Bibliometric analysis is widely recognized as a robust tool for gauging the quality, influence, and authenticity of academic publications (Luukkonen, 1990; Ekinci et al., 2015; Zhao et al., 2018). Moreover, it has been used to evaluate literature in various fields of medical research. For instance, Wu et al., 2022 investigated the interplay between gut microbiota and colorectal cancer using tools such as CiteSpace and VOSviewer. Correspondingly, Yu et al., 2022 used these platforms to gain insights into metabolomics and its connection to CAD, whereas Zhang et al., 2023 mapped key themes and directions in 3D printing applications for cardiovascular ailments. Despite the burgeoning interest in CHD and hyperlipidemia, no bibliometric analysis focusing on lipid-lowering treatments for combined CHD and hyperlipidemia utilizing both VOSviewer and CiteSpace has been conducted yet. This study aimed to fill this gap by dissecting research hotspots and prospective research avenues within the field of lipid-lowering therapy for this combined condition. Through a comprehensive analysis of article formats, publications trends, journal prominence, keyword evolution, and collaborative networks of authors and institutions, this study sought to reveal the current state and future directions of the field, enhancing our understanding of lipid-lowering therapies for CHD and hyperlipidemia and actively contributing to the advancement of research in this area.

## 2 Materials and methods

### 2.1 Search strategy

After obtaining relevant title keywords and supplementing them with grid subject headings from PubMed, we conducted a search using the Web of Science database (Core Collection) as the data source, following the search format shown below: ((ALL=((coronary heart disease) OR (coronary artery disease) OR (coronary disease) OR (silent myocardial ischemia) OR (angina) OR (stenocardia) OR (myocardial infarction) OR (ischemic cardiomyopathy) OR (ischemic heart disease) OR (coronary death) OR (acute coronary syndrome) OR (CHD) OR (CAD) OR (ACS)) AND ALL=((total cholesterol) OR (triglyceride) OR (high-density lipoprotein cholesterol) OR (low-density lipoprotein cholesterol) OR (non-high-density lipoprotein cholesterol) OR (very low-density lipoprotein) OR (intermediate-density lipoprotein) OR (apolipoprotein A1) OR (apolipoprotein B) OR (lipids) OR (lipid metabolism) OR (fatty acid) OR (phospholipid) OR (lipoprotein))) AND ALL=((fibrates) OR (fibric acid) OR (statins) OR (ezetimibe) OR (niacin) OR (Omega-3) OR (proprotein convertase subtilisin-kexin type 9 inhibitor) OR (evolocumab) OR (alirocumab) OR (inclisiran)). The search covered the period from 1 January 1986, to 31 December 2023, and was performed on 31 December 2023.

### 2.2 Study selection

**FIGURE 2:**
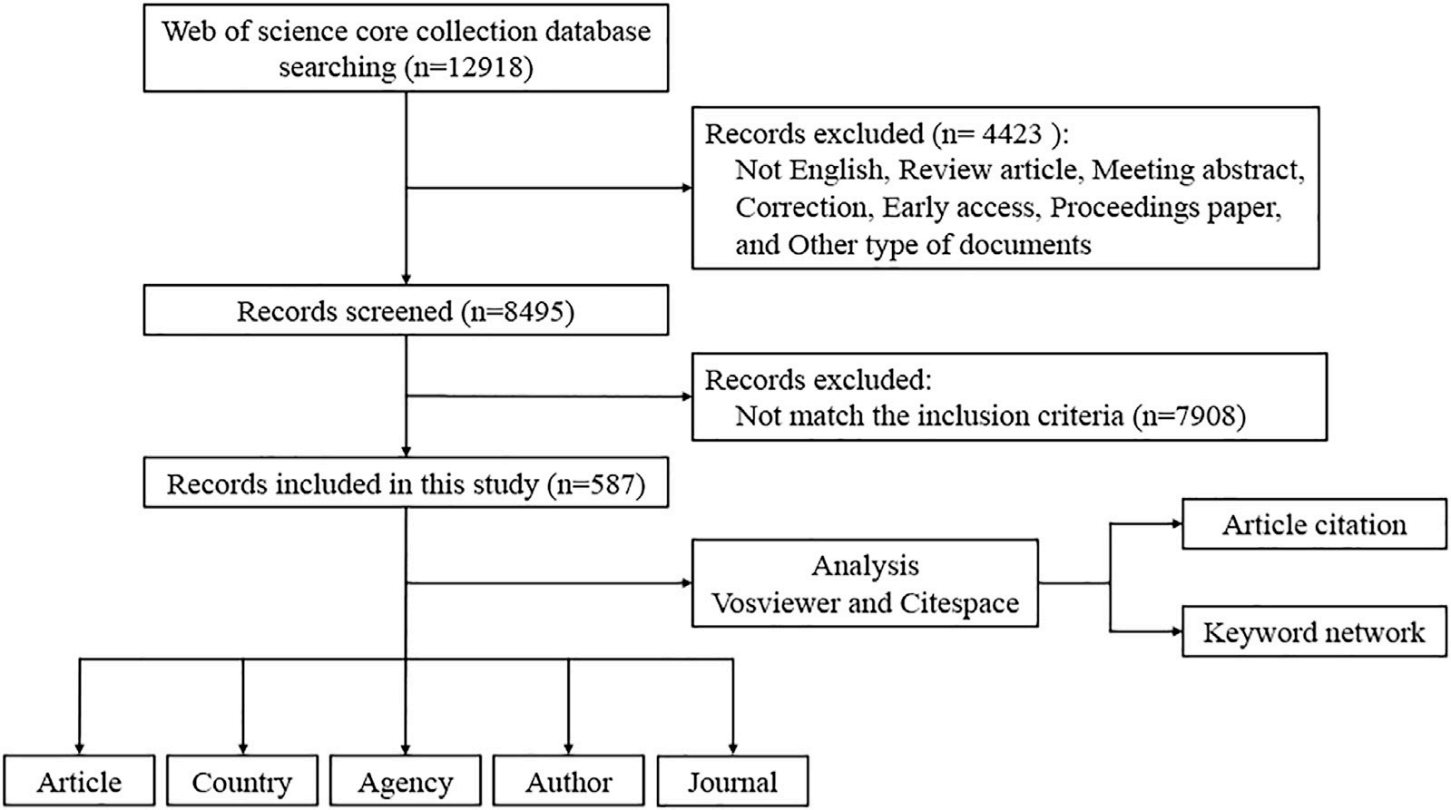
Process of including and excluding relevant publications from research in the field.

Figure 2 outlines the methodological screening process and the precise inclusion criteria used in this study. The search was initiated using the predefined terms described in the previous section. To mitigate potential bias resulting from database updates, two independent researchers reviewed the initially identified publications on the same day, ensuring the validity of the assessment process. During this thorough review, publications that did not meet our eligibility criteria were removed, resulting in a final total of 587 papers. The inclusion criteria were as follows: 1) publications on lipid-lowering therapy for treating CHD with hyperlipidemia, including studies of patients with CHD combined with hyperlipidemia and animal models of CHD with hyperlipidemia (hyperlipidemia included simple hypercholesterolemia and hypertriglyceridemia), that assessed the correlation between lipid-lowering therapies and disease; 2) studies published from 1 January 1986, to 31 December 2023, to ensure the inclusion of publications from 2023; 3) publications from the Web of Science (Core Collection) Citation Index Expanded (SCI-E) and Social Science Citation Index (SSCI) databases; 4) studies published as articles; and 5) studies published in the English language. The exclusion criteria were as follows: 1) reviews, meta-analyses, conference abstracts, letters, early access papers, conference proceedings, corrections, retractions, and other types of publications; 2) duplicate publications; and 3) publications with incomplete bibliographic information, such as title, country, authors, keywords, or source (Gao et al., 2023; Wang et al., 2023).

### 2.3 Data acquisition

We meticulously extracted comprehensive data from literature records, including the titles, authors, abstracts, keywords, and cited references of the publications. These records were then exported into various file formats to facilitate in-depth analysis. During this analytical phase, we evaluated several key bibliometric indicators, including the total number of publications, distribution of publications by country, citation counts, H-index, and detailed information on contributing countries, institutions, authors, journals, and prevalent keywords (Hirsch, 2007).

### 2.4 Data statistics

The imported data were further analyzed using VOSviewer (version 1.6.20) and CiteSpace (version 6.1.6) software. VOSviewer is particularly adept at mapping collaborations among authors, institutions, and countries/regions through co-citation networks. It generates collaborative network maps in which each node, represented as a dot, signifies a country, region, institution, or author. The publication count determines the node size, and the thickness of the connecting lines indicates the collaboration strength. This visualization facilitates a comprehensive understanding of the global research landscape (van Eck and Waltman, 2010). VOSviewer can also be used for keyword cluster analysis, in which frequently co-occurring keywords are grouped into distinct clusters, each marked with a unique color. The resulting graphic reveals prevailing research themes and trends (van Eck and Waltman, 2017). Use of the program Pajek enabled further refinement of cluster layouts to improve clarity and distinction. CiteSpace software specializes in literature co-citation analysis and tracks the evolution of keyword trends over time. Its graphical representation includes nodes symbolizing individual research articles, connected by lines that vary in color and width. These variations denote the strength of collaborations, with darker and thicker lines indicating stronger collaborations (Chaomei, 2005). CiteSpace also examines keyword frequency and identifies “keyword bursts,” crucial for pinpointing emerging research directions (Chen et al., 2012; Chen et al., 2014).

## 3 Results

### 3.1 Analysis of publication numbers, citation trends, and H-index by year

The database search yielded 12,918 records published between 1 January 1986, and 31 December 2023. From this pool, 4,423 were non-research papers and were therefore excluded. The screening process, involving both abstract and full-text reviews, refined the selection to a total of 587 papers. To mitigate potential bias from database updates, a final search was conducted on 31 December 2023, as illustrated in Figure 2.

**FIGURE 3:**
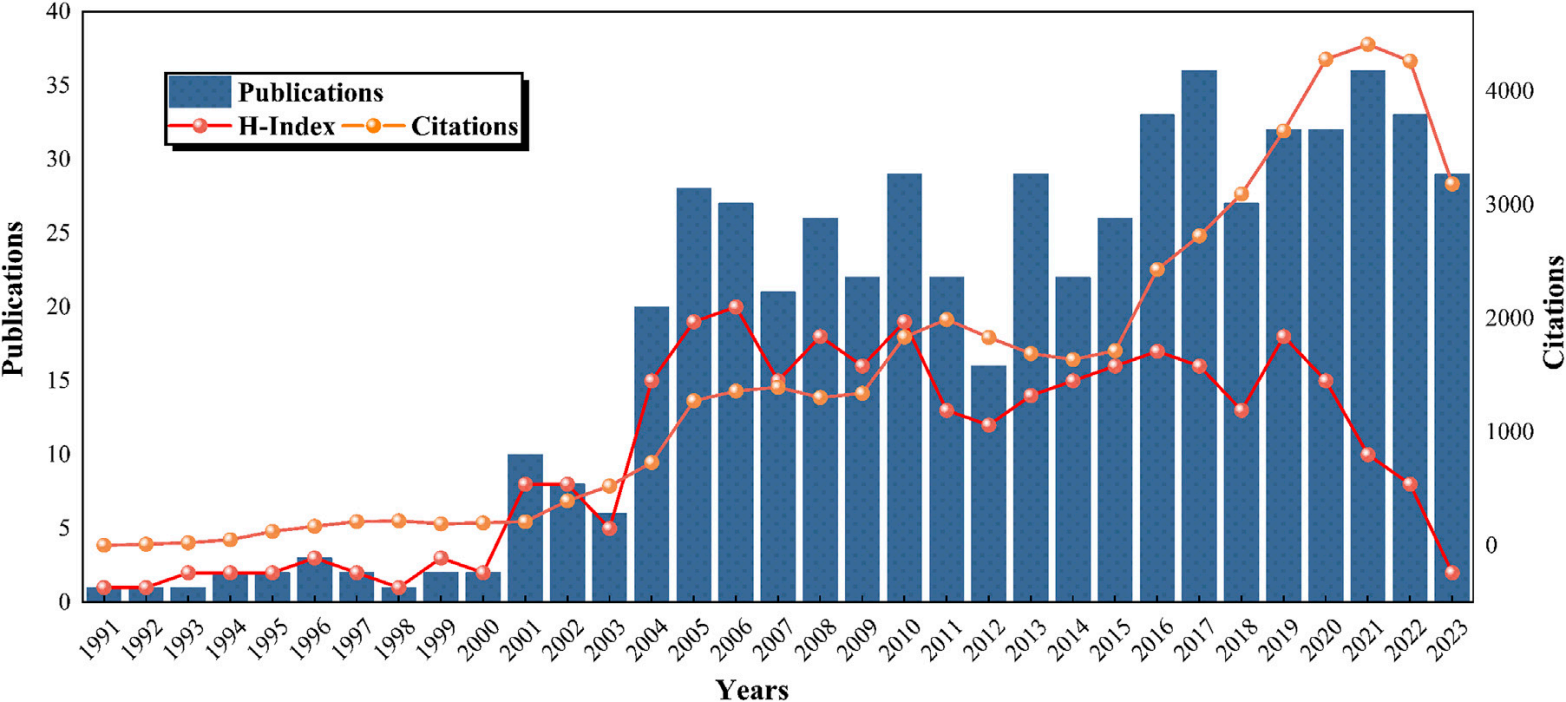
Global trends in publications, citations, and publication H-index in the field of lipid-lowering therapy for CHD combined with hyperlipidemia (1991–2023).

Analysis of annual publication numbers revealed an initial phase (1991–2003) characterized by a modest and stable output. A publication surge occurred between 2003 and 2015, indicating a significant increase in research productivity. The period after 2015 showed a further increase in publications, laying a robust foundation for ongoing research (Figure 3). The trend analysis from 1991 to 2023 indicates a generally ascending trajectory in publication numbers, surpassing 20 in 2004 and 30 in 2016, with occasional dips, including in 2012 and 2018. The number of citations reached its peak in 2021, surpassing 4,400. The H-index of the field remained <10 until 2003 and then experienced a rapid ascent after 2004, peaking at 15–20 between 2005 and 2010. A gradual decline was observed from 2011 to 2018, with a notable decrease post-2019, potentially due to indexing delays. These patterns underscore the increasing interest in lipid-lowering therapies for CHD and hyperlipidemia and project a sustained positive trajectory in research output.

### 3.2 Analysis of journals

**FIGURE 4:**
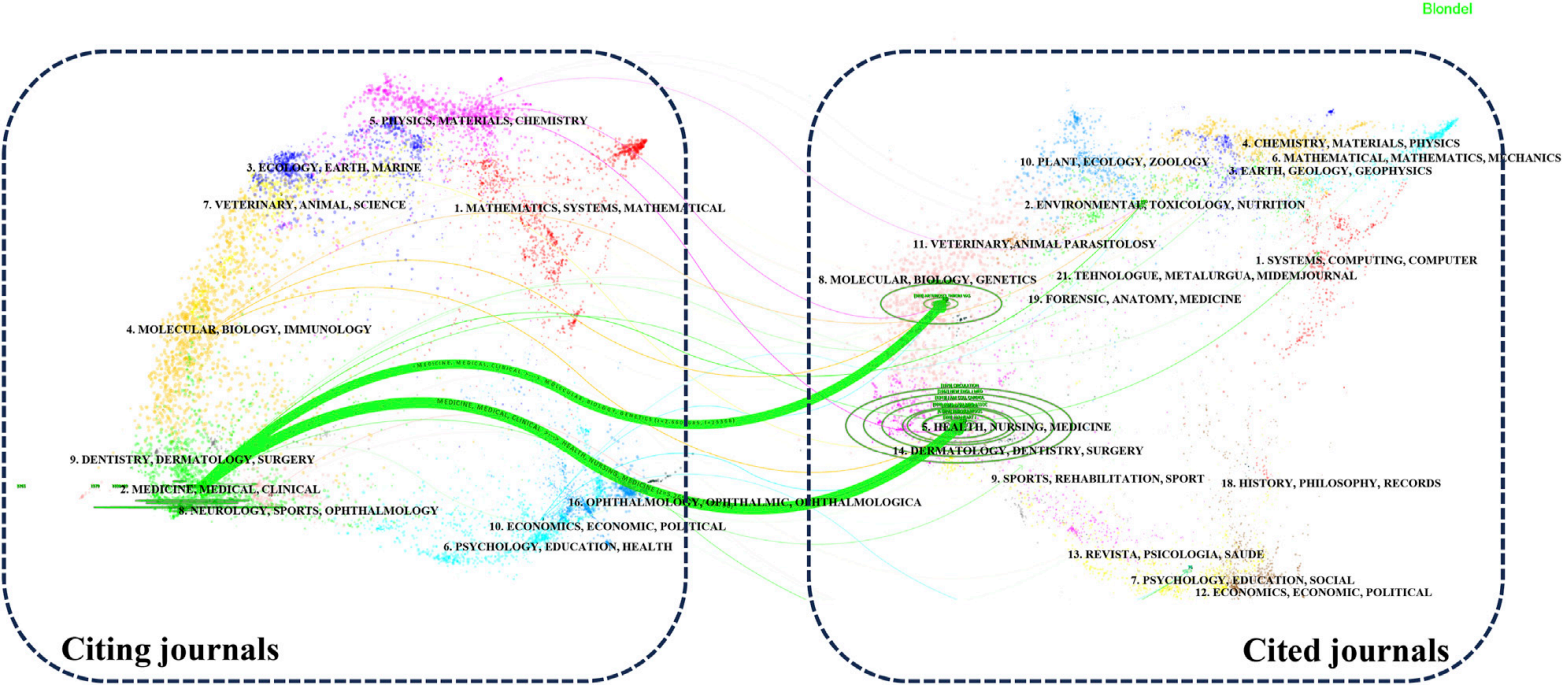
The dual-map overlay of journals.

Our analysis of the publication landscape revealed that as of December 2023, 184 SCI-indexed journals featured articles on lipid-lowering therapy in the context of CHD co-occurring with hyperlipidemia. Table 1 displays the top 10 journals, accounting for 43.10% of the total publications in this area. The American Journal of Cardiology published the highest number of articles (40%, 6.81%), closely followed by Circulation. In terms of citations, Circulation had the highest number (6,266 citations), followed by the Journal of the American College of Cardiology. Circulation, although ranking second in publication volume, had the highest average citation count per article (179.03), an H-index of 33, and an impact factor (IF) of 39.92. These findings highlighted the significant influence of cardiovascular researchers in this field. Figure 4 illustrates the disciplinary focus of the journals involved, with citing journals on the left and cited journals on the right. The analysis revealed a prevailing publication trend within the “medicine, medical, clinical” discipline for research on lipid-lowering therapies in CHD with hyperlipidemia. Moreover, the citation impact of these publications is primarily attributed to journals within the “molecular, biology, genetics” and “health, nursing, medicine” disciplines, revealing a multidisciplinary interest and emphasizing the foundational role of these areas in advancing lipid-lowering therapeutic research.

**TABLE 1:** The top 10 leading journals in the field of lipid-lowering therapies for coronary heart disease combined with hyperlipidemia from 1991 to 2023.

Journal	Publications	Citations	Citations per-publication	H-index	Journal IF (2023)
American journal of cardiology	40	2024	50.6	25	2.80
Circulation	35	6266	179.03	33	39.92
Journal of the American college of cardiology	27	2985	110.56	21	3.13
Atherosclerosis	25	941	37.64	17	6.85
Circulation journal	24	924	38.5	19	3.30
European heart journal	21	1530	72.86	18	35.85
International journal of cardiology	17	621	36.53	14	3.50
Journal of cardiology	16	339	21.19	10	2.50
American heart journal	13	1119	86.08	12	5.09
journal of clinical lipidology	13	185	14.23	9	4.40
Current medical research and opinion	11	303	27.55	10	2.70
Journal of atherosclerosis and thrombosis	11	89	8.09	5	4.40

### 3.3 Analysis of countries/regions

**FIGURE 5:**
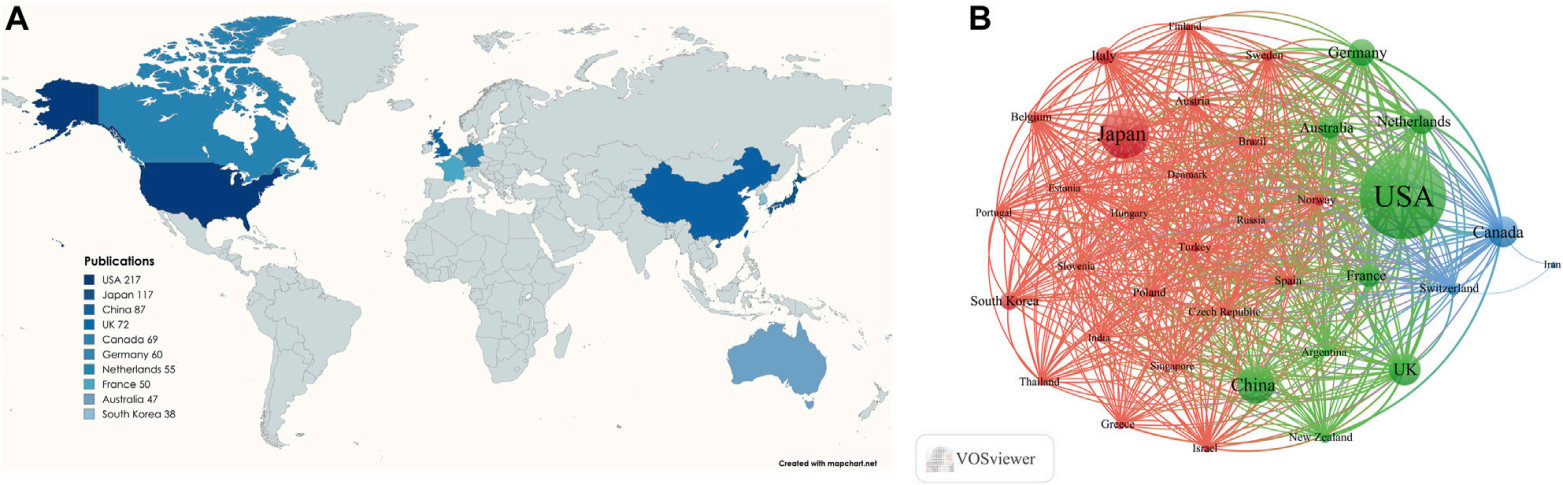
**(A)** Visualization of the number of the top 10 countries in terms of cumulative number of publications; **(B)** Cooperation networks in countries/regions all over the world.

A total of 68 countries have contributed to research on lipid-lowering therapies for CHD combined with hyperlipidemia. Figure 5A presents a map indicating the 10 countries with the highest number of publications, and Table 2 provides detailed information on publications from these countries. The highest number of publications originated from the United States (*n* = 217%, 26.72%), followed by Japan (*n* = 117%, 14.41%), and China (*n* = 87%, 10.71%). The United States also had the highest H-index (69), followed by Germany (38), and the highest number of total citations (*n* = 32,782), followed by Canada (*n* = 17,179), and the UK (*n* = 16,031). Cooperation between the 36 countries with ≥10 publications is depicted in Figure 5B. The United States, Japan, and China are the most central countries in the network, with the United States being the most prominent. China has fewer connections with other countries and should, therefore, intensify efforts to enhance communication and cooperation with the international community.

**TABLE 2:** The top 10 most productive countries/regions in the field of lipid-lowering therapies for coronary heart disease combined with hyperlipidemia from 1991 to 2023.

Countries/Regions	Publications	H-index	Citations	Citations per-publication	Total link strength
United States of America	217	69	32,782	151.07	632
Japan	117	35	5836	49.88	245
China	87	19	2970	34.14	284
United Kingdom	72	37	16,031	222.65	461
Canada	69	36	17,179	248.97	405
Germany	60	38	10,983	183.05	394
Netherlands	55	37	11,221	204.02	408
France	50	32	8355	167.1	409
Australia	47	35	10,678	227.19	366
South Korea	38	16	2498	65.74	230

### 3.4 Analysis of institutions

**FIGURE 6:**
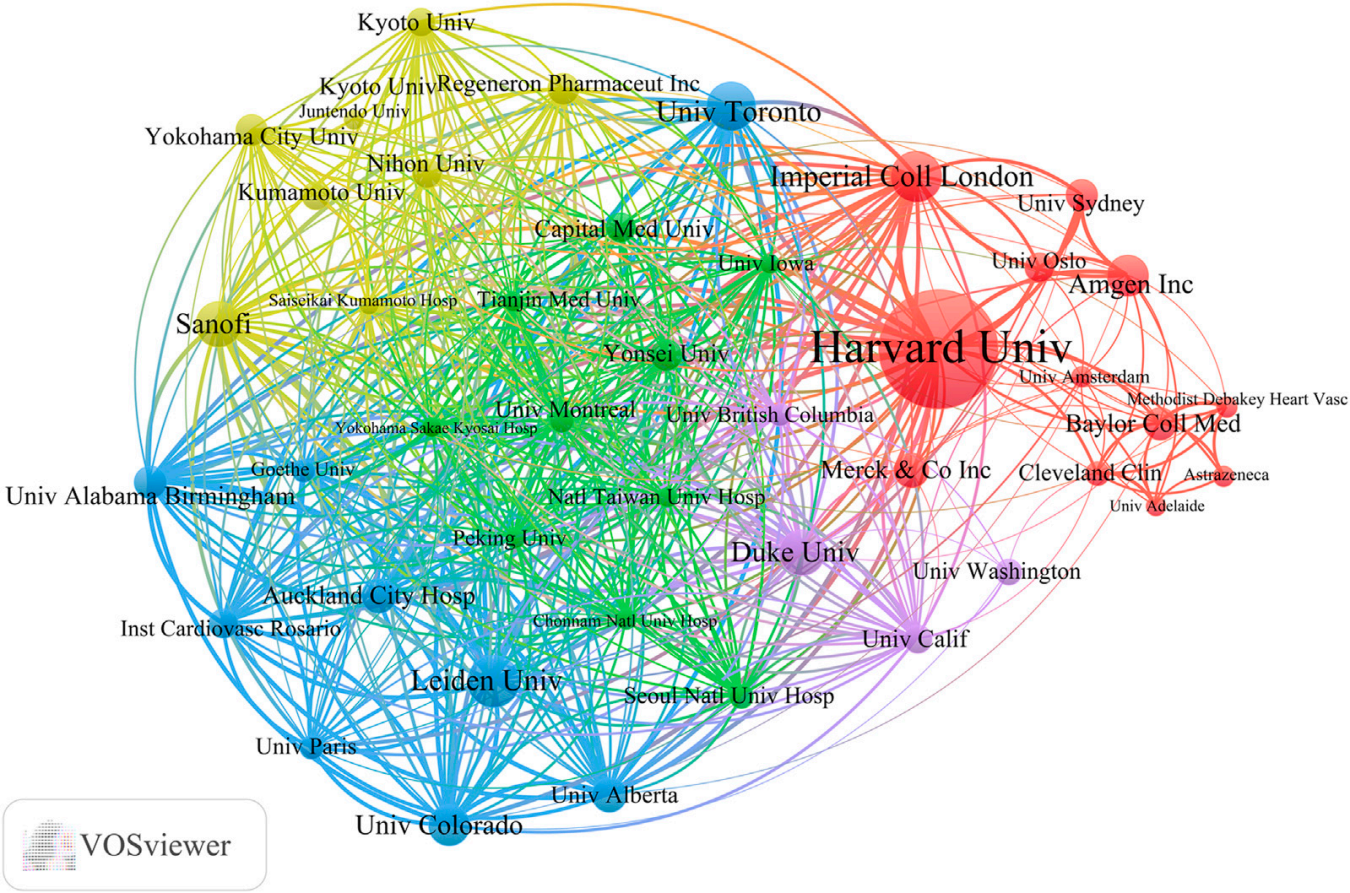
Collaborative network of universities/institutions all over the world.

Globally, 2,805 universities and institutions have contributed to the advancement of lipid-lowering therapies for CHD in the context of hyperlipidemia. Table 3 outlines the top 10 contributors based on research output. Harvard University had the most publications (75), followed by Leiden University. Harvard University also possesses the highest H-index (43) and the highest total citation count (22,002). Its average citation rate per article was 293.36, which was second only to Imperial College London. Among these leading institutions, five are located in the United States. Figure 6 provides a visualization of the key institutions and their collaborative networks. Harvard University is a central node, indicating extensive collaboration, whereas Peking University is the most connected Chinese institution. This illustration accentuates the predominance of international collaborations over domestic ones, highlighting the global nature of research efforts in developing lipid-lowering therapies for patients with CHD and hyperlipidemia.

**TABLE 3:** The top 10 most productive universities/institutions in the field of lipid-lowering therapies for coronary heart disease combined with hyperlipidemia from 1991 to 2023.

Institutions	Publications	H-index	Citations	Citations per-publication	TLS
Harvard University	75	43	22,002	293.36	333
Leiden University	30	26	4042	134.73	311
Imperial College London	29	26	9686	334.00	315
University Of Toronto	28	22	2754	98.36	286
Duke University	26	22	3575	137.50	252
Sanofi Aventis	26	19	4376	168.31	294
Amgen	23	17	6282	273.13	71
University Of Colorado	23	17	2523	109.70	296
Auckland City Hospital	19	15	2464	129.68	280
Merck Sharp & Dohme	19	13	1007	53.00	21

### 3.5 Analysis of authors

**FIGURE 7:**
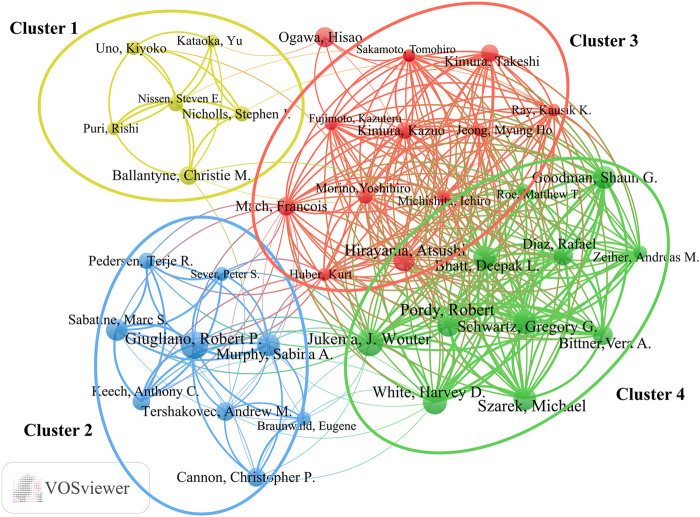
Collaborative network of authors all over the world.

More than 5,900 researchers have contributed to publications in the field of lipid-lowering therapies for CHD and hyperlipidemia. As summarized in Table 4, noteworthy authors include J. Wouter Jukema from the Netherlands and Robert P. Giugliano from the United States, each with 22 publications. Jukema had the highest H-index at 21, whereas Giugliano had the most citations, at 8,825. The collaborative network graph created using VOSviewer in Figure 7 features 38 authors with ≥10 publications, presented in four clusters anchored by key figures such as Jukema, Hirayama, Giugliano, and Ballantyne. Clusters 3 and 4 exhibited strong connections, whereas clusters 1 and 2 displayed fewer interactions, highlighting the variation in collaboration within the research community.

**TABLE 4:** The top 10 most productive scholars in the field of lipid-lowering therapies for coronary heart disease combined with hyperlipidemia from 1991 to 2023.

Author	Publications	Citations	Citations per-publication	H-index	TLS
Jukema, J. Wouter	22	4874	221.55	21	208
Giugliano, Robert P	22	8825	401.14	18	96
White, Harvey D	19	932	49.05	15	208
Schwartz, Gregory G	19	925	48.64	15	204
Goodman, Shaun G	18	2362	131.22	15	212
Szarek, Michael	18	925	51.39	15	204
Bhatt, Deepak L	18	2793	155.17	13	196
Pordy, R	18	2605	144.72	13	195
Murphy, Sabina A	18	5029	279.39	16	75
Hirayama, Atsushi	17	2675	157.35	14	120

### 3.6 Analysis of co-cited references and reference bursts

Table 5 presents the 10 most influential papers in CHD and hyperlipidemia lipid-lowering therapy research based on Web of Science citations. The 2004 study by Cannon et al. in the New England Journal of Medicine leads with 3,617 citations; this study emphasized the advantages of early and substantial LDL-C reduction through statins in patients with ASCVD (Cannon et al., 2004). An earlier work in the same journal by Brown et al. underscored the combined efficacy of simvastatin and niacin in patients with low HDL levels (Brown et al., 2001). Nine out of these 10 pivotal articles originated from institutions in the United States, underscoring the pivotal role of the United States in this research domain, and they were predominantly published in the New England Journal of Medicine (Brown et al., 2001; Cannon et al., 2004; Nicholls et al., 2011; Robinson et al., 2015a; Cannon et al., 2015; Sabatine et al., 2017; Schwartz et al., 2018; Bhatt et al., 2019) or JAMA (Nissen et al., 2004; Nicholls et al., 2016).

**TABLE 5:** The top 10 most cited articles in the field of lipid-lowering therapies for coronary heart disease combined with hyperlipidemia from 1991 to 2023.

Rank	First author	Journal	Title	Citations of Web of science	Institutions
1	Cannon, Christopher P	New England Journal of Medicine 2004; 350 (15):1495–1504	Intensive *versus* moderate lipid lowering with statins after acute coronary syndromes	3617	Icahn School of Medicine at Mount Sinai, Harvard University, The United States
2	Sabatine, Marc S	New England Journal of Medicine 2017; 376 (18):1713–1722	Evolocumab and Clinical Outcomes in Patients with Cardiovascular Disease	3307	Brigham and Women’s Hospital Heart and Vascular Center, Harvard Medical School, The United States
3	Cannon, Christopher P	New England Journal of Medicine 2015; 372 (25):2387–2397	Ezetimibe Added to Statin Therapy after Acute Coronary Syndromes	2719	Icahn School of Medicine at Mount Sinai, Harvard University, The United States
4	NissenSE.	JAMA 2004; 291 (9):1071–1080	Effect of intensive compared with moderate lipid-lowering therapy on progression of coronary atherosclerosis - A randomized controlled trial	1762	Department of Cardiovascular Medicine, Cleveland Clinic Foundation, The United States
5	Bhatt, Deepak L	New England Journal of Medicine 2019; 380 (1):11–22	Cardiovascular Risk Reduction with Icosapent Ethyl for Hypertriglyceridemia	1692	Brigham and Women’s Hospital Heart and Vascular Center, Harvard Medical School, The United States
6	Schwartz, G. G	New England Journal of Medicine 2018; 379 (22):2097–2107	Alirocumab and Cardiovascular Outcomes after Acute Coronary Syndrome	1571	University of Colorado Anschutz Medical Campus School of Medicine, University of Colorado, The United States
7	Robinson, Jennifer G	New England Journal of Medicine 2015; 372 (16):1489–1499	Efficacy and Safety of Alirocumab in Reducing Lipids and Cardiovascular Events	1486	The University of Iowa College of Public Health, University of Iowa, The United States
8	BrownBG.	New England Journal of Medicine 2001; 345 (22):1583–1592	Simvastatin and niacin, antioxidant vitamins, or the combination for the prevention of coronary disease	1483	University of Washington Department of Medicine, University of Washington, The United States
9	Nicholls, Stephen J	JAMA 2016; 316 (22):2373–2384	Effect of Evolocumab on progression of coronary disease in statin-treated patients the GLAGOV randomized clinical trial	682	South Australian Health & Medical Research Institute (SAHMRI) University of Adelaide, The Commonwealth of Australia
10	Nicholls, Stephen J	New England Journal of Medicine 2011; 365 (22):2078–2087	Effect of Two Intensive Statin Regimens on Progression of Coronary Disease	625	Department of Cardiovascular Medicine, Cleveland Hospital, The United States

**FIGURE 8:**
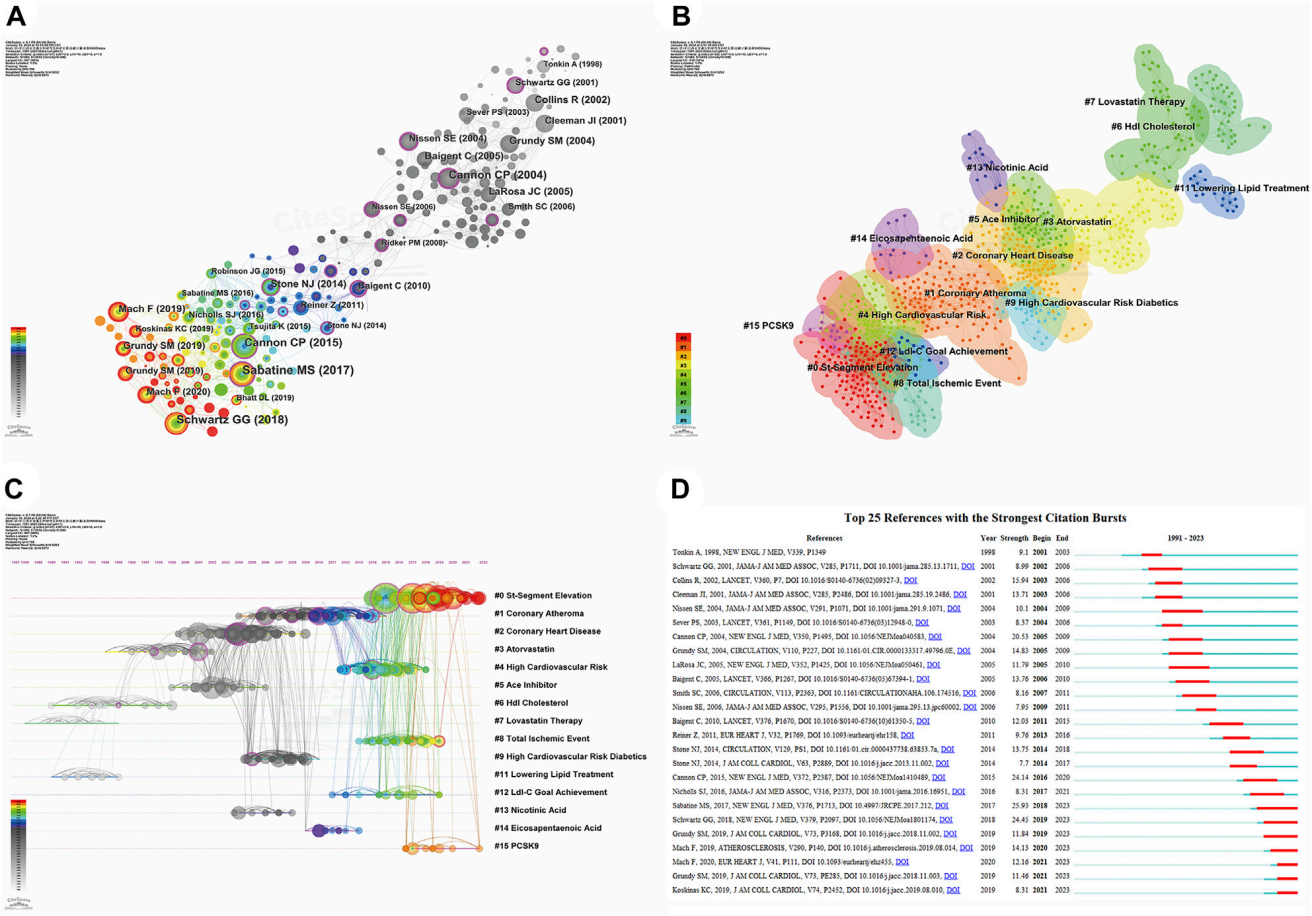
Analyzing reference co-citation through CiteSpace. **(A)** View of reference co-citations; **(B)** Cluster view of reference co-citation; **(C)** Timeline view of reference co-citation. Nodes closer to the right indicate a newer year of citation; **(D)** Top 25 references with the strongest citation bursts. Red bars indicate high citation frequency, blue bars indicate lower citation frequency.

We conducted a co-citation analysis of references using CiteSpace, with the distribution network of co-cited references spanning 1991–2023 presented in Figure 8A. Publications cited >14 times were included in the graphic, with 30 articles highlighted. The color intensity of bars and nodes indicates the publication date, with lighter colors representing more recent citations. Our log-likelihood ratio algorithm identified 14 clusters (Figure 8B), with the largest labeled “St-segment elevation” (# 0), followed by “coronary atheroma” (# 1). We utilized the modularization Q-value criterion to assess the significance of the cluster structure. Typically, when Q-max ≥0.3, the community structure is deemed significant (Wu et al., 2021). In our study, the modularity Q was 0.798, exceeding the threshold of 0.3, and the weighted average profile S was 0.9262, surpassing 0.7. These results indicate that our clustering is robust, convincing, and exhibits homogeneity. Figure 8C provides a timeline view of the reference co-citation clusters, where distinct colored nodes in the same row represent different years of citation in the cluster. Nodes closer to the right represent more recent citations. Figure 8D displays the 25 publications with the strongest citation bursts, with red bars indicating high citation frequency and blue bars indicating lower citation frequency (Zhiguo et al., 2023). The first citation burst in this field occurred in 2001 and lasted 3 years. The study with the strongest burst intensity (strength = 25.93) was published by Sabatine et al., 2017, in 2017 and highlighted the benefits of evolocumab (a PCSK9 inhibitor) in patients with ASCVD. The publications ranked second and third in burst intensity examined the effects of alirocumab (a PCSK9 inhibitor) in patients with ASCVD (strength = 24.45) (Schwartz et al., 2018) and the combination of ezetimibe with statins in patients with ASCVD (strength = 24.14) (Cannon et al., 2015). The greater the intensity of the burst, the more profound its impact on subsequent research.

### 3.7 Keyword visualization

**FIGURE 9:**
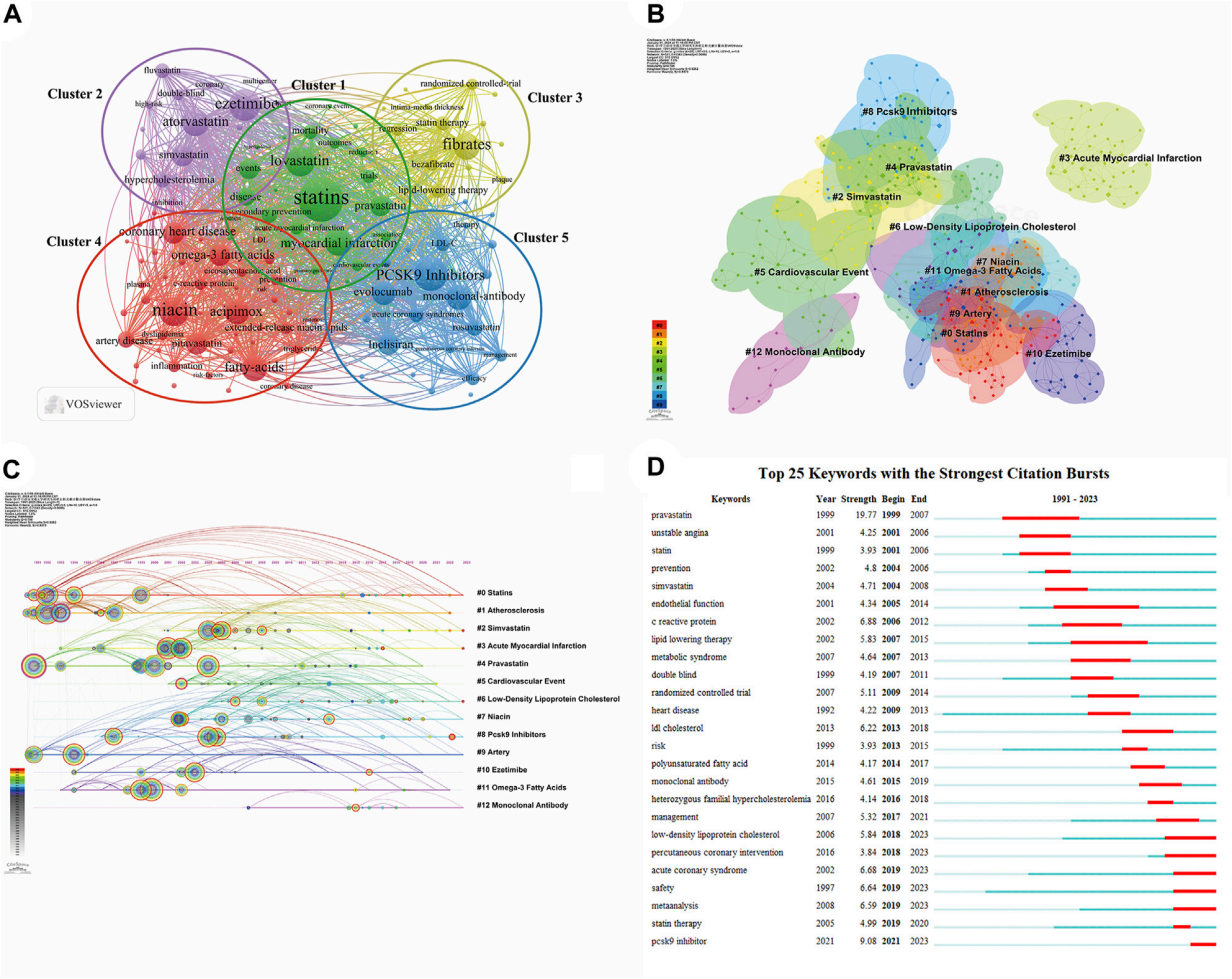
Visualization maps related to studies of lipid-lowering therapy for CHD combined with hyperlipidemia from 1991 to 2023. **(A)** The cooperation network of keywords in the field. The selected keywords can be broadly categorized into five clusters representing different types of lipid-lowering drugs and their combination applications; **(B)** Keyword clustering analysis in this field. Different colors represent different clusters, the smaller the count, the more keywords are in the cluster; **(C)** Timeline view of keywords in the field. The position of a node on the horizontal axis indicates the moment when it first appears, and its size is related to the number of references; **(D)** The top 25 keywords with the strongest citation bursts. The blue bar indicates the time period in which the keyword appeared, and the red bar indicates the start year, end year, and duration of the burst.

Keywords reflect the core content of an article, and analysis of their co-occurrence can identify hot topics and active research areas. We conducted keyword analysis using VOSviewer with a threshold of 8. After combining recurring keywords and synonyms, we obtained 123 keywords. As illustrated in Figure 9A, the selected keywords can be broadly categorized into five clusters, with the most prominent keywords in each cluster being “statins” (Lee S. H. et al., 2023; Meng et al., 2023), “atorvastatin” (Cannon et al., 2004; Nissen et al., 2004), “fibrates” (Räber et al., 2022a; Lee et al., 2023b; Lee et al., 2023c; Kovach et al., 2023), “niacin” (Bonaca et al., 2018; O'Donoghue et al., 2019; Nakao et al., 2023), and “PCSK9” (Lou et al., 2022; Park et al., 2023; Schwartz et al., 2023). The five different clusters represent different lipid-lowering drugs and their combination, suggesting that these may be potential research hotspots. CiteSpace was used to perform cluster analysis on the generated keywords, as depicted in Figure 9B. A total of 13 clusters were formed, with the two most active being “statins” (# 0) and “atherosclerosis” (# 1). Most of the clusters and their synonyms can also be found in the co-occurrence analysis, such as “omega-3 fatty acids” (# 0), “atherosclerosis” (# 1), “simvastatin” (# 2), “pravastatin” (# 4), “PCSK9” (# 8), and “ezetimibe” (# 10). Figure 9C provides a timeline view of keyword clustering, indicating that research on lipid-lowering therapeutic drugs and strategies has persisted from 1991 to the present day, with a major focus on the period up to 2005. After 2005, novel drugs and therapeutic strategies using PCSK9 inhibitors and monoclonal antibodies gained popularity as a research topic. Figure 9D displays the top 25 keywords in terms of outbreak intensity, revealing that “pravastatin” had the strongest intensity (strength = 19.77) and was initiated first (1999). Current active keywords include “low-density lipoprotein cholesterol,” “acute coronary syndrome,” “safety,” and “PCSK9 inhibitor,” indicating emerging research avenues.

## 4 Discussion

### 4.1 Sources and distribution of publications

Before the dawn of the 21st century, the primary focus of lipid-lowering therapy for CHD in conjunction with hyperlipidemia was statins and fibrates. Research by Dairou highlighted the mechanism by which hydroxymethylglutaryl (HMG) CoA reductase inhibitors or statins curbed intracellular cholesterol production while promoting the expression of LDL-C receptors, effectively reducing LDL-C levels (Dairou, 1994). During this period, the United States emerged as a leader in the field, providing the first publications and maintaining the highest publication output to the present day. This leading position may be primarily attributed to the substantial investment in statins research and development by pharmaceutical giants such as Pfizer, Merk Sharp & Dohme (MSD), and Amgen. In particular, MSD’s development of Lovastatin, the first-generation drug for reducing LDL-C levels and the first to be approved for use in the United States, propelled the use and development of this class of drugs worldwide (Blankenhorn et al., 1991).

In the early 2000s, research on lipid-lowering therapies for CHD and hyperlipidemia began to have broader global participation. The field experienced a significant surge in publications starting in 2003, with key years such as 2004 and 2017 marked by substantial increases in publications, citations, and H-indexes, probably largely driven by the discovery of PCSK9 and the development of PCSK9 inhibitors. The American company Amgen significantly impacted this area by developing the first-generation PCSK9 inhibitor, evolocumab, which continues to be widely used. This increased the attention on innovative lipid-lowering agents and strategies. Despite broader international engagement, the top contributors remain primarily from Europe, North America, and Asia, with China as the leading developing country. Collaboration and output analysis highlighted strong ties among developed nations, with the United States, led by Harvard University, playing a pivotal role in shaping the research landscape. International cooperation is an important component of the development of advanced lipid-lowering treatments for CHD and hyperlipidemia.

Chinese scholars began publishing articles in this field in 2004 (Morales et al., 2004), and the number of publications from China has increased consistently each year to its current global ranking of third. Despite its substantial output, China lags behind other countries in terms of the average number of citations per publication and the H-index, indicating a weaker research impact. This discrepancy may be attributed to the numerous research institutions in China, the high volume of publications, and the dominance of large-scale research by developed countries. Key contributors from China include Prof. Tse Hung Fat from the University of Hong Kong, Prof. Zhou Yujie from Sichuan University, and Prof. Huo Yong from Peking University, who collaborate closely with each other and the international community (Goodman et al., 2019; Jukema et al., 2019; Ray et al., 2019; Steg et al., 2019; Szarek et al., 2019; White et al., 2019). As central figures in this field in China, they are expected to continue to foster cooperation and facilitate knowledge exchange.

Our analysis highlighted J. Wouter Jukema as a leading author in the field, publishing significant randomized controlled trials, epidemiological studies, and various research methods, with a focus on evaluation of the lipid-lowering efficacy of various lipid-lowering drugs in CHD with hyperlipidemia. Robert P. Giugliano, another leading author in the field, uses imaging techniques such as vascular ultrasound to examine arterial plaque composition and stability, assessing the effectiveness of new lipid-lowering drugs or therapies. The collaborative efforts of these two authors significantly influence the direction of the field (Cannon et al., 2015; Bonaca et al., 2018; O'Donoghue et al., 2019). Despite being the foremost publisher by article count, the American Journal of Cardiology did not have the highest IF or number of citations, suggesting a potential compromise between research quality and quantity. Circulation, with its high IF and leading citation metrics, may, therefore, be the more influential journal in this field.

Overall, the global research pattern on lipid-lowering therapies for CHD and hyperlipidemia displays a distinct geographical distribution, with developed nations, notably the United States, leading the field. However, as economic and healthcare standards improve, collaboration between developed and developing countries continues to rise. As a vital developing country, China has distinctly intensified its academic partnerships with developed nations, including the United States, particularly following its involvement in the ODYSSEY OUTCOMES study post-2017. This collaboration resulted in a significant increase in publications from Chinese institutions, which now account for 71.26% of the total research output in this domain, emphasizing the vast potential for cooperation with research institutions in developed countries.

### 4.2 Development and research trend of lipid-lowering therapeutic drugs and strategies

Our study identified five keyword clusters, as illustrated in Figure 9A. Each cluster represents a distinct drug development and therapeutic strategy for CVDs, with a shift in focus from clusters 1 to 5.

#### 4.2.1 Cluster 1 (green): statins

Cluster 1 represents the pioneering role of statins, particularly lovastatin, the first statin drug developed and brought to market by MSD, in treating CHD and hyperlipidemia. This cluster marks the inception of CVD prevention and treatment through lipid-lowering strategies, laying the groundwork for the development of new lipid-lowering medications and therapies. The well-established link between cholesterol levels and atherosclerotic heart disease emphasizes the critical role of LDL-C elevation and HDL-C reduction (Kannel et al., 1979; Keys et al., 1984; Brown et al., 2006; Vergallo and Crea, 2020). Statistically, a 1 mg/dL reduction in LDL-C is associated with a 1% lower risk of cardiovascular events, whereas a 1 mg/dL increase in HDL-C levels corresponds to a 2%–3% risk reduction (Phan and Toth, 2013). Over 3 decades, studies have consistently validated the efficacy of statins, demonstrating not only their ability to slow coronary atherosclerosis progression but also their significant impact on reducing LDL-C levels and the risk of cardiovascular events (Pedersen et al., 1994; Sacks et al., 1996; Collins et al., 2002; Shepherd et al., 2002; Baigent et al., 2005). More recently, two studies have shown that statin use effectively reduces the prevalence of non-alcoholic steatohepatitis and fibrosis in patients with metabolic dysfunction, helping to prevent non-alcoholic fatty liver disease (Ayada et al., 2023; Li et al., 2023). This indicates that statins may also have hepatoprotective effects. The effects of them are likely linked to their ability to inhibit HMG-CoA reductase, reduce LDL-C and triglyceride levels, inhibit the proliferation of vascular smooth muscle, regulate platelet activity, reduce lipid synthesis, and suppress the production of pro-inflammatory cytokines (Paradela-Dobarro et al., 2013; Madasamy et al., 2016; van Rosendael et al., 2021; Ayada et al., 2023). Despite being the focus of early lipid-lowering therapy research, the limitations of statins become evident with disease progression. Low doses may not achieve therapeutic goals, and escalating doses often result in adverse effects such as muscle pain, cognitive issues, and liver and kidney toxicity, compromising treatment efficacy (Toth et al., 2008; Avis et al., 2010; Cohen et al., 2012). In addition, some research has indicated that pregnant women exposed to statins are at an elevated risk of preterm delivery, and this suggests an impact on the cardiovascular health of their offspring later in life (Chang et al., 2021; Wu et al., 2023). Therefore, developing more effective and safer lipid-lowering drugs and therapies may improve patient outcomes.

#### 4.2.2 Cluster 2 (purple): combination of statins and ezetimibe

Cluster 2 centers on the synergistic use of ezetimibe, a cholesterol absorption inhibitor, and statins to address hyperlipidemia. Ezetimibe was jointly developed by MSD and Schering-Plough in the United States in 2002. It functions by selectively blocking the NPC1L1 protein in the intestinal tract, thereby inhibiting cholesterol absorption and reducing the transport of cholesterol to the liver. This effectively lowers blood LDL-C and total cholesterol levels. This pioneering selective cholesterol absorption inhibitor has been shown to achieve a 20% reduction in LDL-C levels (Bruckert et al., 2003).

The dual-action mechanism of ezetimibe hinders dietary cholesterol absorption and stimulates endogenous cholesterol production in the liver without negatively affecting triglyceride levels (Nutescu and Shapiro, 2003). When combined with statins, ezetimibe enhances the treatment of resistant hyperlipidemia or dyslipidemia, significantly decreasing LDL-C levels and boosting the effectiveness of lipid-lowering therapies (Stein et al., 2004). Gagné et al. demonstrated that combining 10 mg of ezetimibe with either 40 or 80 mg of a statin led to a 20.7% reduction in LDL-C levels. Due to its minimal drug interactions, ezetimibe is suitable for patients with low statin tolerance (Gagné et al., 2002). This breakthrough led to the development of other novel treatments for patients experiencing adverse effects from statin treatment.

#### 4.2.3 Cluster 3 (yellow): combination of statins and fibrates

Cluster 3 focuses on the use of fibrates, either alone or in conjunction with statins, for lipid-lowering and plaque stabilization. Although statins have been the cornerstone of lipid-lowering treatment, particularly for patients with hyperlipidemia and CHD, their effectiveness in slowing atherosclerosis progression diminishes over time for high-risk patients. Research indicates that doubling the statin dose achieves only a 6% reduction in LDL-C levels and a 22% decrease in cardiovascular event risk, with high TG levels remaining a residual risk factor (Ray and Cannon, 2004; Ray and Cannon, 2006; Liew and Ray, 2008). As early as the late 1960s, the American pharmaceutical company Pfizer developed the first fibrate drug, gemfibrozil, intended for the treatment of pure hypertriglyceridemia. Fibrates, derived from phenoxyaromatic acid, effectively lower serum TG levels and elevate HDL-C levels by activating peroxisome proliferator-activated receptor alpha and lipoprotein lipase; they also confer anti-inflammatory and antithrombotic benefits (Perreault et al., 2011; Tenenbaum and Fisman, 2012; Gross et al., 2017; Kim and Kim, 2020). Clinical trials have demonstrated the significance of fibrates in treating atherosclerotic dyslipidemia, confirming their role as a potent option for patients with elevated TG levels (Brunner et al., 2000; Robins et al., 2001; Scott et al., 2009; Elam et al., 2017). Over the following 15 years, the Germany pharmaceutical company Bayer and the French pharmaceutical company Sanofi each developed their own fibrates, Fenofibrate and Bezafibrate, respectively. Fenofibrate continues to be a first-line medication for the treatment of patients with high TG and LDL-C levels.

#### 4.2.4 Cluster 4 (red): combination of statins, niacin, and omega-3 fatty acids

Cluster 4 represents the combined use of niacin, omega-3 fatty acids, and statins for treating CHD with hyperlipidemia. Niacin, similar to fibrates, reduces TG levels and increases HDL-C levels. Kim et al. demonstrated that niacin activates sirtuin-1, enhancing mitochondrial fatty acid oxidation and reducing TG synthesis (Kim et al., 2010). Although combining niacin with statins impacts TG and HDL-C levels, a resultant significant reduction in the risk of cardiovascular events remains to be demonstrated (Bruckert et al., 2010; Boden et al., 2011; Haynes et al., 2013).

Omega-3 fatty acids, including eicosapentaenoic acid, docosahexaenoic acid, and docosapentaenoic acid, are recognized for their polyunsaturated nature and pharmacological significance (Calder, 2018). The earliest related medication was developed by Pronova BioPharma and approved for use in the United States in 2004. Omega-3 fatty acids lower the levels of TRLs and promote the production of anti-inflammatory and anticoagulant mediators, contributing to thrombosis resistance and atherosclerotic plaque stabilization (Larsson et al., 2004; Nordestgaard, 2016a). Although omega-3 fatty acids have been shown to decrease CHD mortality in high-risk patients (Mozaffarian and Wu, 2011), definitive evidence linking them to a significant reduction in major adverse cardiovascular events (MACE) in this population remains lacking (Aung et al., 2018). Thus, further research on the synergistic effects of niacin and omega-3 fatty acids on MACE, coupled with the optimization of dosing strategies, presents new avenues for investigation in this sector.

#### 4.2.5 Cluster 5 (blue): combination of statins and PCSK9 inhibitors

Cluster 5 shows the synergistic use of statins with PCSK9 inhibitors to enhance ASCVD prevention efficacy.

PCSK9, a serine protease produced by liver cells, binds to LDL receptors, hindering their recycling and diminishing liver capacity to clear LDL-C. This, in turn, increases the risk of hyperlipidemia and the development of ASCVD (Abifadel et al., 2003; Piper et al., 2007; Urban et al., 2013; Lin et al., 2018). PCSK9 inhibitors have proven effective in significantly reducing LDL-C levels and cardiovascular risks (Hadjiphilippou and Ray, 2017). PCSK9 inhibitor monotherapy has been shown to achieve a >50% reduction in LDL-C levels compared with intensive statin treatments (Hadjiphilippou and Ray, 2017; Räber et al., 2022b), and more pronounced coronary plaque regression is observed when their use is combined with high-intensity statins (Schwartz et al., 2018).

In contrast, inclisiran, a small interfering RNA analog developed by Novartis in 2019, targets the PCSK9 protein in hepatocytes to disrupt RNA transcription and translation, reducing plasma LDL-C levels (Nair et al., 2014; Dyrbuś et al., 2020). Recent ORION trials have demonstrated the ability of inclisiran to substantially reduce LDL-C levels by 47.9% compared to placebo in patients with heterozygous familial hypercholesterolemia (Raal et al., 2020). Additionally, inclisiran demonstrated an even more pronounced reduction of 52.3% in patients unresponsive to other lipid-lowering medications (Ray et al., 2020). Fitzgerald et al. found that adverse reactions to inclisiran were primarily limited to coughing, headaches, and diarrhea, indicating an overall favorable safety profile (Fitzgerald et al., 2017). The findings from the ORION-7 study were consistent with these results, reporting no serious adverse reactions (Wright et al., 2020). Although inclisiran holds significant promise as a lipid-lowering therapy, direct evidence linking it to a reduction in MACE remains elusive. Future research should, therefore, prioritize investigating the impact of inclisiran on MACE while ensuring its ongoing safety.

### 4.3 Contribution of pharmaceutical companies to lipid-lowering drug research

Pharmaceutical companies play a crucial role in the research and development of lipid-lowering medications. Through their efforts, they have significantly advanced the treatment of hyperlipidemia and related CVDs. Supplementary Table S1 provides details of the 10 pharmaceutical companies with the highest number of publications. Since MSD pioneered the development of lovastatin in 1987, statins have revolutionized the management of high cholesterol levels and significantly reduced the risk of cardiovascular events for millions of patients worldwide. The success of lovastatin led to the development of a series of other statins. From 1991 to 2003, companies such as Sankyo, MSD, Novartis, Pfizer, and AstraZeneca introduced pravastatin, simvastatin, fluvastatin, atorvastatin, and rosuvastatin. These developments established the United States as a world leader in this field and also represented significant progress for Japan. As the demand for CVD treatments intensified at the beginning of the 21st century, the emergence of complex hyperlipidemia spurred the development of other lipid-lowering medications. Sanofi and Amgen introduced alirocumab and evolocumab, respectively, which were designed to be used in conjunction with statins to further reduce lipid levels (Kühnast et al., 2014; Robinson et al., 2015b). Companies such as MSD, Sanofi, Regeneron Pharmaceuticals, Amgen, AstraZeneca, and Pfizer have played pivotal roles in advancing the development and application of lipid-lowering drugs, which is consistent with the results shown in our table. However, despite being one of the top three pharmaceutical companies in the world, Novartis ranks at the bottom, which we suspect is due to the recent introduction and limited uptake of inclisiran. The development of new targets for lipid-lowering therapies is also accelerating. For example, clinical trials for drugs targeting antisense oligonucleotides (ASO) and angiopoietin-like protein 3 (ANGPTL3) are currently being conducted by companies such as Ionis Pharmaceuticals and Regeneron Pharmaceuticals. As research progresses, these companies will continue to play a crucial role in advancing lipid management to prevent CHD.

### 4.4 Hotspots and frontiers in lipid-lowering therapy

Keyword analysis highlighted the research hotspots and core content in this field; however, understanding the latest lipid-lowering treatments in hyperlipidemia and CHD is equally important. Recent research has proposed several unconventional treatment methods, such as cuproptosis therapy, endoplasmic reticulum stress inhibition therapy, and therapies involving H_2_S or SO_2_. A study published by Yang et al. noted an increased presence of copper ions within atherosclerotic plaques; however, an excessive deficiency of copper ions can elevate cholesterol levels (Yang et al., 2023). These seemingly contradictory findings may be attributed to the impact of copper ions on endothelial inflammatory factors. They underscore the need for a balanced concentration of copper ions to effectively reduce the risk of atherosclerosis and hyperlipidemia (Málek et al., 2000; Habas and Shang, 2018). Furthermore, research by Keylani et al. indicated that endoplasmic reticulum stress is closely linked to the progression of atherosclerosis and lipid metabolism disorders, a phenomenon attributed to its induction of specific inflammatory responses (Huang et al., 2018; Keylani et al., 2022). Atorvastatin improves atherosclerosis and reduces cellular damage by inhibiting inflammatory responses (Yang and Hu, 2015). Meanwhile, a study by Song et al. describes a different approach, finding that sirtuin-1 improves atherosclerosis by regulating the acetylation of certain functional proteins, and treatment with H_2_S enhances its expression, deacetylation function, and stability, ultimately inhibiting the development and progression of CHD (Du et al., 2019; Song et al., 2023).

**FIGURE 10:**
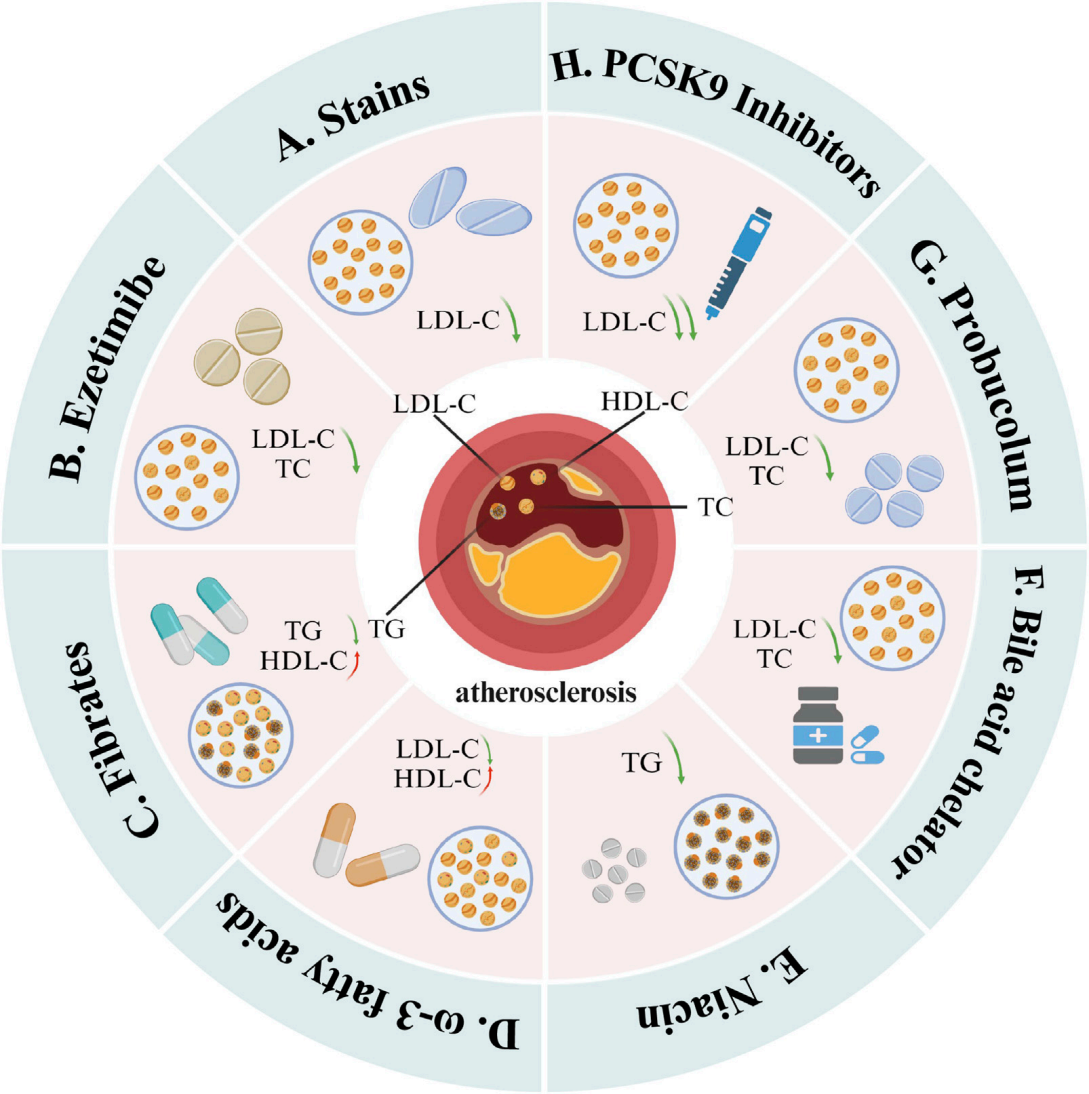
Mainstream drugs that can be used for lipid-lowering treatment. These include **(A)** statins, **(B)** ezetimibe, **(C)** fibrates, **(D)** ω-3 fatty acids, **(E)** niacin, **(F)** bile acid sequestrant, **(G)** antioxidant and **(H)** PCSK9 inhibitors. Among them, statins mainly reduce LDL-C levels; ezetimibe, bile acid sequestrants and antioxidants mainly reduce TC and LDL-C levels; fibrates and niacin mainly reduce TG levels, but fibrates also It has the effect of increasing HDL-C; ω-3 fatty acids can increase HDL-C levels and reduce LDL-C levels; PCSK9 inhibitors can significantly reduce LDL-C levels (Created with BioRender.com).

Despite these innovative treatment methods, the use of lipid-lowering drugs remains the primary strategy for treating hyperlipidemia and CHD. Current research continues to seek the optimal combination therapy for managing these conditions. Figure 10 depicts several commonly used lipid-lowering medications: statins, ezetimibe, fibrates, omega-3 fatty acids, niacin, bile acid sequestrants, antioxidants, and PCSK9 inhibitors. Among these, statins continue to form the cornerstone of lipid-lowering therapy. Their continued popularity is due not only to their significant clinical efficacy but also to the extensive research underlying their use and their multi-system therapeutic effects.

The advent of PCSK9 inhibitors marks a significant advancement in pharmacological lipid-lowering therapy, particularly for the treatment of primary hypercholesterolemia and the prevention of cardiovascular events. Evolocumab and alirocumab, as the first-generation PCSK9 inhibitors, are now widely used in patients with poor LDL-C control and acute coronary syndrome. The FOURIER trial conducted by O'Donoghue et al. demonstrated that long-term use of evolocumab can maintain LDL-C levels below 40 mg/dL, with good safety and tolerability profiles; furthermore, early treatment with evolocumab significantly reduced the risk of adverse cardiovascular events (O'Donoghue et al., 2022). Studies by Gencer et al. and Wiviott et al. confirmed this finding and indicated that evolocumab treatment results in a greater reduction in absolute risk in patients who recently experienced a myocardial infarction and are, therefore, at a higher risk of MACE (Gencer et al., 2020; Wiviott et al., 2020). For patients with CHD, the use of evolocumab significantly reduces the volume of atherosclerotic plaques and offers improved safety compared to the placebo group (Nicholls et al., 2016). Similarly, a *post hoc* analysis of the ODYSSEY OUTCOMES trial indicated that for patients who have recently been diagnosed with acute coronary syndrome, for whom the accumulation of metabolic risk factors is associated with an increased incidence of MACE, treatment with alirocumab reduces the incidence of MACE and demonstrates good tolerability across various subgroups (Ostadal et al., 2022).

The recently launched second-generation PCSK9 inhibitor, inclisiran, is still in the development phase. Although phase III clinical trials, ORION-10 and ORION-11, have further confirmed that it significantly lowers LDL-C levels, studies such as ORION-4, VICTORION-1 PREVENT, and VICTORION-2 PREVENT, which focus on the reduction of MACE rates, are yet to be completed. Additionally, due to its high treatment costs, inclisiran has not been widely adopted in current practice. However, with its unique advantage of requiring only biannual dosing, inclisiran should significantly improve patient compliance, and it may, therefore, have an important role to play in lipid management and CHD. Furthermore, it is worth noting that the research and development of third-generation PCSK9 inhibitors, such as MK-0616 and lerodalcibep, are actively underway. These are currently in phase III clinical trials and the post-research stage, respectively. The gradual publication of cardiovascular outcome studies and real-world research for these drugs should provide more comprehensive and detailed scientific evidence for their clinical use.

Finally, new-generation lipid-lowering drug targets, such as ASO and ANGPTL3, are gradually emerging. Drugs targeting these molecules are currently in phase II and III clinical trials, respectively, although further in-depth research is required. We expect that the publication of these trial results will further refine the treatment of dyslipidemia, ultimately reducing the risk of ASCVD events.

### 4.5 Advantages and limitations

This study represents a pioneering effort to chart the evolution, current trends, and emerging hotspots in lipid-lowering therapy for patients with CHD and hyperlipidemia. Utilizing tools such as VOSviewer, CiteSpace, and Pajek provides a comprehensive overview of the progression of the field, fosters a deeper understanding of research, and offers insights into notable scholars and their collaborative networks.

Nevertheless, this study has some limitations. First, it encompasses only literature from the SCI-E and SSCI indices in the Web of Science database, potentially overlooking studies in other databases. However, the Web of Science database is globally recognized for its extensive coverage and the high quality of its indexed journals, ensuring access to authoritative research (Zhang et al., 2020a; Zhang et al., 2020b). Furthermore, this study was confined to English language publications, potentially limiting its scope. Additionally, despite the search being conducted on 31 December 2023, newly published articles may not have been included. Finally, the selection of search terms might have introduced some bias; for example, the omission of certain lipid-lowering drugs or other terms for CHD and hyperlipidemia might have led to the exclusion of a small number of relevant studies. However, this bias is acceptable because our study aimed to elucidate the overall framework and development trajectory of research into lipid-lowering therapies for CHD in conjunction with hyperlipidemia. In future research, the comprehensiveness of our conclusions can be enhanced by expanding the list of search terms, extending the range of data sources, and incorporating a variety of analytical indicators (Wang et al., 2023).

## 5 Conclusion

Through bibliometric analysis, this study comprehensively mapped the landscape of lipid-lowering therapy research over the past 40 years, identifying influential countries, institutions, and authors. The notable surge in publications since 2003 underscores the widespread adoption of lipid-lowering therapy as the primary approach for treating and preventing CHD with hyperlipidemia. The evolution of therapeutic approaches reveals a distinct shift towards cutting-edge lipid-lowering agents, particularly PCSK9 inhibitors, and a strategic inclination towards combining these novel agents with statins or other drug classes. In the future, we anticipate a strong research emphasis on novel PCSK9 inhibitors and the development of safe and effective combination therapies. Future research endeavors should focus on pivotal areas such as “PCSK9 inhibitors,” “inclisiran,” “combination therapy,” “safety,” “ASO,” and “ANGPTL3”.

## Data Availability

The original contributions presented in the study are included in the article/Supplementary Material, further inquiries can be directed to the corresponding author.
