# Supplementary material for: Research trends in lipid-lowering therapies for coronary heart disease combined with hyperlipidemia: a bibliometric study and visual analysis
Source: Front Pharmacol. 2024 May 17;15:1393333. doi: 10.3389/fphar.2024.1393333 (PMC11140088; doi:10.3389/fphar.2024.1393333)
Supplement: Supplementary file 1 [file Table1.DOCX]

Supplementary Material

**Supplementary Table 1** The top 10 globally renowned pharmaceutical companies in the field of lowering lipid levels for coronary heart disease combined with hyperlipidemia from 1991-2023

| **Pharmaceutical companies** | **Publications** | **H-index** | **Citations** | **Citations per-publication** | **Country** |
| --- | --- | --- | --- | --- | --- |
| Merk Sharp & Dohme | 34 | 21 | 4887 | 143.74 | The United States |
| Sanofi | 29 | 20 | 4880 | 168.28 | France |
| Regeneron Pharmaceuticals Inc | 25 | 20 | 4777 | 191.08 | The United States |
| Amgen Inc | 24 | 18 | 6844 | 285.17 | The United States |
| AstraZeneca | 14 | 12 | 1280 | 91.43 | The United Kingdom |
| Pfizer Inc | 11 | 10 | 2436 | 221.45 | The United States |
| Roche | 2 | 2 | 150 | 75 | Switzerland |
| Novartis | 1 | 1 | 212 | 212 | Switzerland |
| Eli Lilly and Company | 1 | 1 | 61 | 61 | The United States |
| Abbott | 1 | 1 | 5 | 5 | The United States |
